# Foraging in a dynamic environment: Response of four sympatric sub‐Antarctic albatross species to interannual environmental variability

**DOI:** 10.1002/ece3.6766

**Published:** 2020-09-21

**Authors:** Tegan Carpenter‐Kling, Ryan R. Reisinger, Florian Orgeret, Maëlle Connan, Kim L. Stevens, Peter G. Ryan, Azwianewi Makhado, Pierre A. Pistorius

**Affiliations:** ^1^ Marine Apex Predator Research Unit (MAPRU) Department of Zoology Institute for Coastal and Marine Research Nelson Mandela University Port Elizabeth South Africa; ^2^ DST‐NRF Centre of Excellence at the FitzPatrick Institute of African Ornithology Nelson Mandela University Port Elizabeth South Africa; ^3^ LOCEAN‐IPSL UMR 7159 CNRS‐IRD‐MNHN Sorbonne Université Paris France; ^4^ Centre d'Etudes Biologiques de Chizé UMR 7372 du CNRS‐Université de La Rochelle Villiers‐en‐Bois France; ^5^ FitzPatrick Institute of African Ornithology DST‐NRF Centre of Excellence University of Cape Town Rondebosch South Africa; ^6^ Department of Environment, Forestry and Fisheries Oceans and Coasts Research Cape Town South Africa

**Keywords:** behavioral plasticity, biologging, central place foragers, marine predators, resilience, Southern Annual Mode

## Abstract

Seasonal and annual climate variations are linked to fluctuations in the abundance and distribution of resources, posing a significant challenge to animals that need to adjust their foraging behavior accordingly. Particularly during adverse conditions, and while energetically constrained when breeding, animals ideally need to be flexible in their foraging behavior. Such behavioral plasticity may separate “winners” from “losers” in light of rapid environmental changes due to climate change. Here, the foraging behavior of four sub‐Antarctic albatross species was investigated from 2015/16 to 2017/18, a period characterized by pronounced environmental variability. Over three breeding seasons on Marion Island, Prince Edward Archipelago, incubating wandering (WA, *Diomedea exulans*; *n* = 45), grey‐headed (GHA, *Thalassarche chrysostoma*; *n* = 26), sooty (SA, *Phoebetria fusca*; *n* = 23), and light‐mantled (LMSA, *P. palpebrata*; *n* = 22) albatrosses were tracked with GPS loggers. The response of birds to environmental variability was investigated by quantifying interannual changes in their foraging behavior along two axes: spatial distribution, using kernel density analysis, and foraging habitat preference, using generalized additive mixed models and Bayesian mixed models. All four species were shown to respond behaviorally to environmental variability, but with substantial differences in their foraging strategies. WA was most general in its habitat use defined by sea surface height, eddy kinetic energy, wind speed, ocean floor slope, and sea‐level anomaly, with individuals foraging in a range of habitats. In contrast, the three smaller albatrosses exploited two main foraging habitats, with habitat use varying between years. Generalist habitat use by WA and interannually variable use of habitats by GHA, SA, and LMSA would likely offer these species some resilience to predicted changes in climate such as warming seas and strengthening of westerly winds. However, future investigations need to consider other life‐history stages coupled with demographic studies, to better understand the link between behavioral plasticity and population responses.

## Graphical abstract

Anomalous climatic events change the predictability of resource distributions; this can have detrimental effects on species foraging efficiency and subsequently their population health. By analyzing GPS tracks of four species of albatross over three years characterized by pronounced climatic variability, we show that these albatrosses cope with variability within their environments using different foraging strategies. This is an encouraging finding as it indicates possible resilience of these species to climate change.

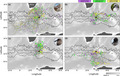

## 1 INTRODUCTION

The spatial distribution of resources and its predictability across time is a major driver of animal movement (Fauchald, 1999; Van Moorter et al., 2013; Mueller et al., 2011; Riotte‐Lambert & Matthiopoulos, 2019; Weimerskirch, 2007). If the distribution of resource patches is static or temporally predictable (e.g., daily, seasonally), specialization in foraging behavior is expected to occur (Futuyma & Moreno, 1988; Pyke, Pulliam, & Charnov, 1977; Switzer, 1993). For example, when animals forage within predictable resource patches, they should exhibit site fidelity, frequenting habitats, or areas that are known to be productive (Arthur et al., 2015; Augé, Chilvers, Moore, & Davis, 2014). In contrast, animals foraging in dynamic landscapes, where the distribution of resources is unpredictable, are expected to exhibit some flexibility in aspects of their foraging behavior (Hastings, 1983; Kassen, 2002). By so doing, they thus optimize the probability of prey encounter by utilizing a variety of foraging habitats (Dehnhard et al., 2019; Gilmour et al., 2018) and/or searching extensively for food resources (Mueller et al., 2011; Weimerskirch, 2007).

The spatio‐temporal distribution of resources across marine environments is determined by multiple scale‐dependent physical and biological processes (Lévy, Jahn, Dutkiewicz, Follows, & d’Ovidio, 2015; Scales et al., 2014). At macro‐ (100–1,000 km) to meso‐scales (10 km), areas of upwelling at prominent topographical features, major oceanic fronts and eddies concentrate prey resources and act as predictable foraging grounds for marine predators (Bost et al., 2009; Scales et al., 2014; Wakefield, Phillips, & Matthiopoulos, 2009; Weimerskirch, 2007). However, environmental variability can alter these complex bio‐physical processes resulting in varying degrees of predictability in resource location across different time scales (Brown et al., 2010; Fraser & Hofmann, 2003; Trathan et al., 2006).

The way marine predators respond to variability in their environments and associated predictability of prey patches varies between life‐history stages, species, and populations (Sydeman, Poloczanska, Reed, & Thompson, 2015). For example, the foraging duration of breeding seabirds is constrained by their need to regularly return and perform nest duties at a central location (Phillips, Croxall, Silk, & Briggs, 2008). Thus, it is expected that due to limited time to search for prey, they would have suitable foraging strategies to cope with potential variability in prey distribution (Riotte‐Lambert & Matthiopoulos, 2019). Indeed, many seabird species respond to climatic variability and associated changes in resource availability by either switching their diet to temporally available prey (Carpenter‐Kling et al., 2019; Machovsky‐Capuska et al., 2018), utilizing different foraging habitats (Cleeland et al., 2019; Geary, Leberg, Purcell, Walter, & Karubian, 2020; Phillips, Hindell, Hobday, & Lea, 2019), or following the distribution of preferred prey (Kappes et al., 2010; Pettex et al., 2012). However, even if they demonstrate such behavioral plasticity, suitable foraging habitats may become inaccessible or energetically costly to reach during climatically anomalous years (e.g., Bost et al., 2015), potentially resulting in breeding failure and/or population declines (e.g., Croxall, Reid, & Prince, 1999; Thorne et al., 2015). Thus, in light of the rapid habitat degradation and increased environmental variability imposed by climate change, the ability of a species to respond has important implications for their foraging efficiency and population trajectories (Cooke, Eigenbrod, & Bates, 2019; Jenouvrier et al., 2018).

The Prince Edward Archipelago, situated in the south‐west Indian Ocean sector of the Southern Ocean, consists of two islands (Marion and Prince Edward islands) and hosts more than five million breeding seabirds and seals (Ryan & Bester, 2008). Important foraging habitats of these predators include eddies (~100 km in diameter with a longevity of months; Durgadoo, Ansorge, & Lutjeharms, 2010; Lutjeharms & Valentine, 1988) and oceanic fronts (~1,000 km in diameter and consistently present but varying in latitudinal position at a monthly scale; Lutjeharms, 1985). To the north of the islands, warm‐core eddies are spawned as the Agulhas Return Current interacts with the sub‐Antarctic (SAF) and subtropical (STF) fronts within the subtropical convergence zone (STCZ; Lutjeharms & Valentine, 1988; Naik et al., 2015). To the south of the islands, the fast‐flowing Antarctic polar front (PF) interacts with the South‐west Indian Ridge at the Andrew Bain Fracture Zone (ABFZ) resulting in upwelling of nutrient‐rich waters and cold core eddy formation (Ansorge & Lutjeharms, 2003; Durgadoo et al., 2010). These major frontal zones (i.e., SAF and PF) and eddies are known to be rich in zooplankton, forage fish, and cephalopods (Koubbi, 1993; Pakhomov & Froneman, 1999; Pakhomov & Perissinotto, 1997) which are common prey items of seabirds (Cherel & Klages, 1998) and seals (de Bruyn, Tosh, Oosthuizen, Bester, & Arnould, 2009; Klages & Bester, 1998). Thus, it is not surprising that grey‐headed albatrosses (GHA) *Thalassarche chrysostoma* (eddies at STCZ and ABFZ; Nel et al., 2001), southern elephant seals *Mirounga leonina* (SAF, PF, and eddies at ABFZ; Massie et al., 2016; Tosh et al., 2015) and king penguins *Aptenodytes patagonicus* (PF; Pistorius et al., 2017), breeding at the Prince Edward Archipelago, all forage at these mesoscale features.

These important foraging habitats of seabirds and seals breeding at the Prince Edward Archipelago are subject to interannual climatic variability. Eddy production in the Southern Ocean correlates to wind stress, indicated by the Southern Annular Mode index (SAM; Morrow, Ward, Hogg, & Pasquet, 2010; Thompson & Wallace, 2000). A positive SAM index indicates the strengthening and contraction of the Southern Hemisphere westerly winds toward Antarctica (Marshall, 2003). During periods of an anomalously positive SAM index, there is a general increase in the occurrence of eddies in the Antarctic and subtropical zones with a lag response of two to three years, although effects differ regionally (Meredith & Hogg, 2006; Morrow et al., 2010). The SAM index has been predicted to become increasingly positive over the next century (e.g., Gillett & Fyfe, 2013). Seabirds respond both positively (e.g., Weimerskirch, Louzao, De Grissac, & Delord, 2012) and negatively (Fay, Barbraud, Delord, & Weimerskirch, 2017; Sauser, Delord, & Barbraud, 2018) to strengthening of the westerly winds within the Southern Ocean and the associated changes in their foraging habitats. In addition, the latitudinal position of the two major ocean fronts (SAF and PF) straddling the archipelago is highly variable and is predicted to migrate southward in response to future changes in climate (Kim & Orsi, 2014; Wilson et al., 2016 but see Chapman, Lea, Meyer, Sallée, & Hindell, 2020, Meijers et al., 2012). The response of marine predators to environmental variability can be quantified using a variety of approaches including stable isotope analysis (e.g., Fort et al., 2010), monitoring of demographic parameters (e.g., Inchausti et al., 2003; Weimerskirch, 2018), and assessing foraging behavior through deployment of tracking instrumentation (e.g., Kappes et al., 2010; Phillips et al., 2019). Here, we investigate variability in foraging behavior of the four albatross species (wandering *Diomedea exulans* (WA), GHA, sooty *Phoebetria fusca* (SA), and light‐mantled *P. palpebrata* (LMSA) albatrosses) breeding on Marion Island over three consecutive years (2015/16 to 2017/18), a period which was characterized by particularly pronounced climatic variability, indicated by a positive SAM index anomaly during 2015 which became increasingly negative toward the end of 2016 (https://legacy.bas.ac.uk/met/gjma/sam.html).

Specifically, we investigate the response of birds to environmental variability by quantifying interannual changes in their foraging behavior along two axes: spatial distribution and habitat use. We propose three possible hypotheses as to how albatross species may respond to variability within their environment:

1. *Faithful hypothesis*: A species has a consistent spatial distribution or uses a particular foraging habitat (such as fronts, eddies, or shelf edge) or a combination of the two, despite environmental variability.
2. *Flexible hypothesis*: Among years, a species switches between two or more discernibly different foraging habitats in response to environmental variability.
3. *Nomad hypothesis*: A species does not have any foraging habitat preference but consistently searches large areas for resources (i.e., spatial distribution will be similar between years, ranging across a wide range of oceanic habitats).

We predicted that the response of a given albatross species to be dependent on the predictability of its preferred foraging habitat (Riotte‐Lambert & Matthiopoulos, 2019). If a species forages within a predictable prey concentrating oceanographic feature, such as a major frontal zone, we expected that the species will remain faithful to this feature with interannual variability in foraging effort. However, if a species prefers to forage within more ephemeral features, such as mesoscale eddies, we expected that it will either exhibit flexible foraging strategies, foraging within areas of highest eddy presence, or be nomadic and search vast areas for suitable foraging grounds.

At other colonies, WA have been shown to be generalist in term of their foraging habitat use (Weimerskirch, 2007). Their foraging strategy is characterized by searching large areas and foraging in multiple foraging habitats during a single foraging trip (Nel et al., 2002; Weimerskirch, 2007). Therefore, we predict that the WA incubating at Marion Island are generalized in their foraging habitat use and have an extensive foraging distribution which will remain consistent among years (Nomad hypothesis). The GHA and LMSA, breeding elsewhere, have been shown to switch between foraging habitats between years, depending on the interannual foraging habitat quality (Phillips, Silk, & Croxall, 2005; Xavier, Croxall, Reid, Trathan, & Rodhouse, 2003). To our knowledge, interannual variability in foraging behavior of the SA has not yet been investigated but we expect similar behavior to that of LMSA. Therefore, in terms of the GHA, SA, and LMSA, we predict that these species will adjust their foraging habitats in response to environmental variability and demonstrate flexibility in foraging behavior (Flexible hypothesis). Alternatively, they may be faithful to a particular foraging habitat and are thus inflexible in terms of their foraging behavior (Faithful hypothesis).

## 2 METHODS

All data analyses were performed in the R statistical environment (R version 3.6.1; R Core Team, 2019).

### 2.1 Location data

Over three consecutive breeding seasons (2015/16, 2016/17, and 2017/18), GPS data loggers (CatLog‐S GPS loggers, Perthold Engineering LLC USA, 50 × 22 × 8 mm, 34 g) were deployed on four albatross species on Marion Island (46°55ʹS; 37°40ʹE)during the incubation period : wandering (WA), grey‐headed (GHA), sooty (SA), and light‐mantled (LMSA) albatrosses (Table S1). Loggers recorded locations at one‐hour interval and were retrieved after one foraging trip. All deployments were within colonies on the south‐east coast of Marion Island and within GHA colonies along an inland ridge on the south side of the island.

Following the removal of erroneous locations using a McConnel speed filter (*trip* package; Sumner, 2016) based on a conservative movement speed threshold of 135 km/hr (Phillips et al., 2008), trip parameters were calculated for each individual foraging trip. Path length (sum of great circular distances between consecutive locations), maximum distance reached (great circular distance to the furthest point reached from the colony), and trip duration (time between departure and return of the bird to its colony) were calculated (*geosphere* and *trip* packages; Hijmans, 2019; Sumner, 2016).

### 2.2 Track segmentation

Foraging activity along a seabird's track is characterized by high sinuosity (i.e., frequent turning) and low flight speeds and can be distinguished from direct and fast transit to and from the colony (Benhamou, 1992; Pinaud & Weimerskirch, 2007). Here, a Bayesian state‐space model was used to segment tracks into two behaviors: foraging and transit (*bsam* package; Jonsen, 2016; Jonsen, Mills Flemming, & Myers, 2005). Specifically, a first‐difference correlated random walk with behavior switching (DCRWS) was implemented separately for each species. The DCRWS model estimates a behavioral state (*b*; range 1–2) for each location based on the speed, turning angle, and move persistence (autocorrelation) within the track, with lower b values indicating transiting behavior and higher values indicating foraging behavior (Jonsen et al., 2005). To segment tracks into either foraging or transiting, GPS locations with *b* < 1.25 were identified as transiting and GPS location with *b* > 1.75 was identified as foraging (Jonsen, Myers, & James, 2007). Prior to further analysis, locations with b values between 1.25 and 1.75 were removed. This resulted in the removal of 24.0% of all locations. Furthermore, all locations recorded at night were removed as they could not readily be distinguished as resting or “sit and wait” foraging (Phalan et al., 2007; Pinaud & Weimerskirch, 2007). Locations within 15 km of the coast of Marion Island were removed to avoid overrepresentation of this area, as albatrosses often preen on the water close to their colonies before departing on a foraging trip (Granadeiro, Campioni, & Catry, 2017).

### 2.3 Environmental data

To investigate the association between the foraging behavior of albatrosses and oceanographic features, five environmental variables were considered: four satellite‐derived measures of altimetry: sea surface height (SSH), sea‐level anomaly (SLA), eddy kinetic energy (EKE), and wind speed, and one static variable: ocean floor slope (Table S2). Sea surface temperature was not used as it is highly correlated to SSH. Environmental variables were extracted for each location identified as foraging or transiting by the DCRWS. A Spearman's correlation matrix revealed that there was no collinearity (coefficient < 0.7) between any of the extracted environmental variables (Dormann et al., 2013).

Within the Southern Ocean, fronts are identifiable by the SSH values (Table S3; Swart, Speich, Ansorge, & Lutjeharms, 2010). SSH values of mesoscale eddies can be identical to values associated with fronts (Lamont, van den Berg, Tutt, & Ansorge, 2019). Thus, to confirm birds’ association with eddies, EKE was also used (Table S2). The SLA was used to investigate association with either cyclonic (<−0.25 m) or anticyclonic (>0.25 m) eddies (see Dragon, Monestiez, Bar‐Hen, & Guinet, 2010). Wind speed, zonal winds, and meridional winds have previously been shown to impact the foraging behavior of albatrosses (Weimerskirch et al., 2012), and thus, they were included within the analysis. However, as the three components are heavily correlated, only wind speed was included as a proxy for all three wind components. Ocean floor slope, calculated as the gradient of bathymetry (*raster* package; Hijmans, 2020), was used to identify bathymetric features such as continental shelves, fracture zones, or sea mounts. EKE and slope values were log transformed to improve homoscedasticity and normality of residuals.

### 2.4 Detection of variability within the marine environment

To assess environment variability during our study period, the following was plotted over a five‐year period (2014–2018). Firstly, monthly SAM indices (https://legacy.bas.ac.uk/met/gjma/sam.html) were plotted. Secondly, monthly eddy activity was calculated for the eddy field in the lee (to the east) of the ABFZ and an area of the STCZ (Ansorge & Lutjeharms, 2003; Naik et al., 2015) by averaging the EKE within two bounding boxes which encompassed these areas (Figure S4). The bounding box to characterize the ABFZ eddy field was chosen following the methods of Asdar (2018), who characterized this eddy field and studied its EKE. The bounding box of the STCZ eddy field was chosen based on this area previously being shown to be of importance to foraging seabirds (Nel et al., 2001) and seals (Massie et al., 2016; Tosh et al., 2015). Thirdly, the mean latitudinal position of the SAF and PF between 35 and 45°E was calculated monthly, following Asdar (2018). Lastly, monthly wind speed averaged over the extent of albatrosses’ tracks was plotted.

### 2.5 Spatial distribution

Interannual differences in trip parameters (i.e., path length, duration, and maximum distance) were investigated using permutational analysis of variance (PERANOVA; *coin* package; Hothorn, Hornik, van de Wiel, & Zeileis, 2008). Within separate models, trip parameters were set as the response variable and species and year and their interaction set as the predictor variable. Where necessary, this was followed by pairwise permutation tests (*rcompanion* package; Mangiafico, 2019) with false discovery rate corrections (Benjamini & Hochberg, 1995).

Intraspecific overlap in spatial foraging distribution between years was investigated using a kernel density analysis (Worton, 1989) based on locations inferred to be where albatrosses were foraging. The geographic locations were transformed to Cartesian coordinates using a Lambert Cylindrical Equal Area projection before utilization distributions (UD) were calculated on a 50‐km grid (*adehabitatHR* package; Calenge, 2006). A species‐specific smoothing parameter (*h*) was calculated by calculating UDs for each individual using the automated *h* selection (“href”) and a species‐specific *h* value taken as the average of these values. The 95% and 50% UD contours were calculated to represent the foraging range and core foraging areas of each species, respectively. Overlap among breeding seasons was calculated using Bhattacharyya's affinity (Bhattacharyya, 1943). The null hypothesis of year UDs not overlapping was tested by permuting track labels between years 1,000 times and calculating the overlap for each permutation. *p*‐Values were estimated as the proportion of times the observed overlap was greater than the permuted overlap.

### 2.6 Foraging habitat preference

Generalized additive mixed models (GAMMs) were used to quantify the correlation between albatross foraging behavior and environmental variables (Wood, 2006). Because outliers can have a strong influence on model fit, an Outlier Test based on an Analysis of Regression in R‐student was used to identify outliers (following the methods of Tew Kai & Marsac, 2010). Specifically, binomial GAMMs with a logit link function were used with individual identity as a random factor, to allow for individual variation (*mgcv* package; Wood, 2006). Behavior, that is, foraging (1) or transiting (0), was set as the response variable and the interactions between all environmental variables and year as predictor variables. Treating the putative behavioral mode of the birds at a given location as the binomial response variable in the GAMMS enabled the comparison of environmental conditions at locations where birds were likely foraging with conditions at locations where they were likely transiting. This allowed for the probability of each species to be in either behavioral state to be assessed as a function of the given environmental variables. Smoothers were fitted to each environmental variable using cubic regression splines with shrinkage to avoid over fitting (Wood, 2006). As a GAMM inference relies upon independence between observations, spatial correlation between observations was accounted for by incorporating a smooth longitude by latitude interaction term in models (Cleeland et al., 2019; Dormann, 2007). Smoothing splines were estimated via restricted maximum likelihood (REML). Year was included as a categorical variable in the fixed effects part of each model using the “by” argument to the gam function in the *mgcv* package, allowing smooths to be generated for each environmental variable for each year. To test whether including year produced a more parsimonious model, the Akaike information criterion (AIC) resulting from models including and excluding year were compared and the model with the lowest AIC value was selected (Pedersen, Miller, Simpson, & Ross, 2019). In terms of the environmental variables, model selection was performed using best subset selection (James, Witten, Hastie, & Tibshirani, 2013), which involves building models with all possible combinations of predictor terms and selecting the best model as the one with the lowest AIC value. Separate models were built for each species. For all species, models which included year and all environmental variables resulted in the lowest AIC values, and thus, only models including year are reported in the results. In addition, concurvity among environmental variables was tested for and was found to be below 0.5 for all variables. To allow for comparison between species, fitted values were back transformed to probability using the *plogis* function.

### 2.7 Variability in foraging habitat

Interannual variance in foraging habitat use by individuals of different species was investigated using multivariate Bayesian mixed models (*MCMCglmm* package; Hadfield, 2010). Only values of the environmental variables at foraging locations were included as a multivariate response variable within models and were scaled with a z‐transformation. Year and species were included as a fixed effect to allow for between‐year and between‐species variation and individual ID nested within species as a random intercept to account for repeat measures within individuals. To estimate a per species and year variance value for each environmental variable, a heterogenous residual variance structure was fitted with species variance nested within year. Models with and without a heterogenous residual variance structure and fixed effect were compared, with the best model being selected based on the lowest deviance information criterion (DIC) score. All models including a heterogenous residual variance structure and fixed effects were found to have lower DIC scores than their counterparts. Visual checks for posterior distributions and autocorrelation were used to ensure convergence.

## 3 RESULTS

**FIGURE 1:**
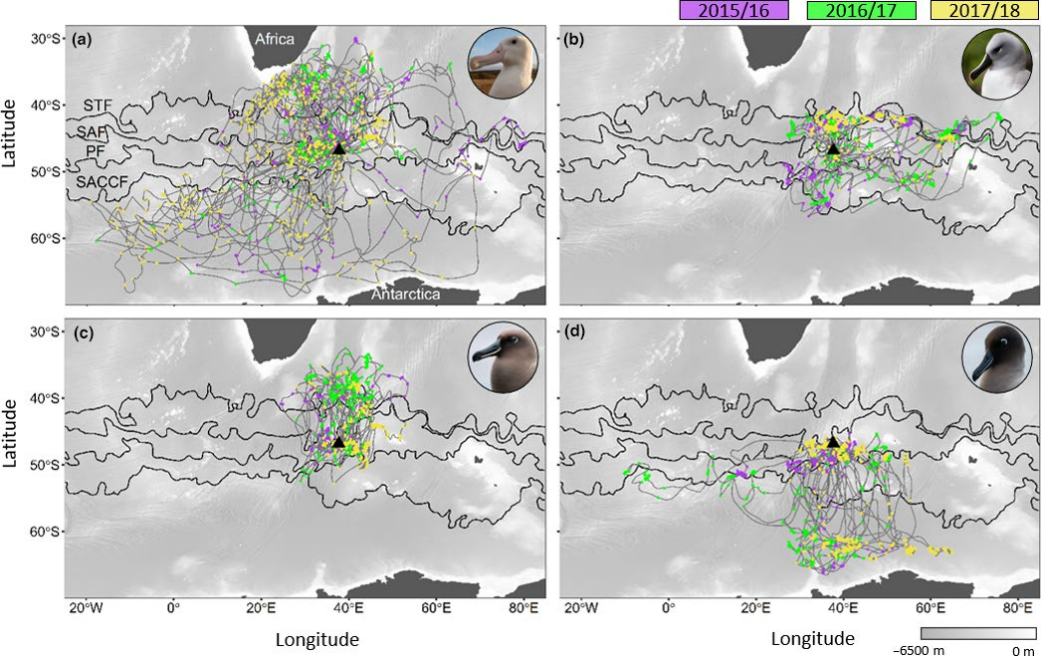
Tracks of (a) wandering (*n*_2015/16_ = 9, *n*_2016/17_ = 16, *n*_2017/18_ = 20), (b) grey‐headed (*n*_2015/16_ = 5, *n*_2016/17_ = 11, *n*_2017/18_ = 10), (c) sooty (*n*_2015/16_ = 5, *n*_2016/17_ = 10, *n*_2017/18_ = 8), and (d) light‐mantled (*n*_2015/16_ = 5, *n*_2016/17_ = 6, *n*_2017/18_ = 11) albatrosses incubating on Marion Island (black triangle) over three consecutive breeding seasons. Points show inferred foraging locations along the tracks of the albatrosses, each year is denoted by a different color:purple = 2015/16, green = 2016/17, and yellow = 2017/18, in relation to the position of subtropical (STF), Sub‐Antarctic (SAF), Antarctic Polar (PF), and Southern Antarctic Circumpolar (SACCF) fronts

Over the three years, 116 complete foraging trips of incubating albatrosses were recorded, 45 trips for WA, 26 trips for GHA, 23 trips for SA, and 22 trips for LMSA (Figure 1; Table S1). These trips covered an extensive area, ranging from Africa to Antarctica (Figure 1). The distribution of the WA covered this area, with evidence of foraging across all frontal zones (Figure 1a). In contrast, the three smaller albatrosses had more restricted foraging ranges and exhibited directed travel toward areas where they switched to a foraging behavioral mode (Figure 1b–d, Figure S5).

### 3.1 Environmental variability

**FIGURE 2:**
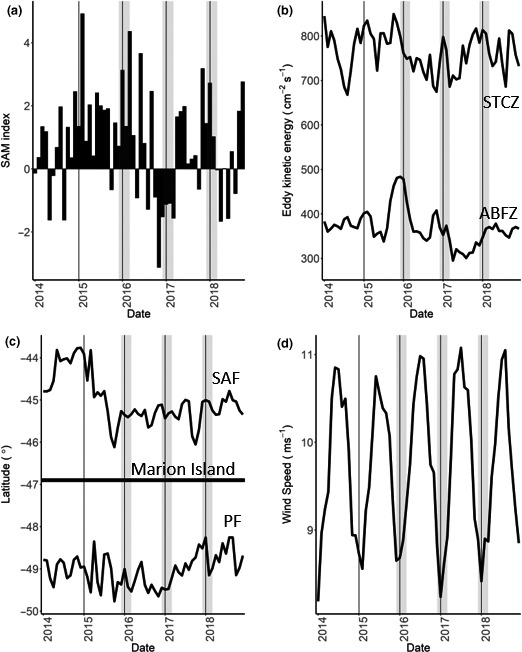
Monthly values of the (a) Southern Annular Mode (SAM) index, (b) monthly mean eddy kinetic energy of an area of the Sub‐Tropical Convergence Zone (STCZ) and Andrew Bain Fracture Zone (ABFZ) eddy fields, (c) mean latitude position of the Sub‐Antarctic (SAF) and Antarctic Polar (PF) fronts between 35 and 40°E, black horizontal line shows the latitude of Marion Island, and (d) monthly wind speeds averaged over the extent of the albatross tracks. Vertical solid black lines indicate the beginning of each year. Gray‐shaded boxes indicate months albatrosses were tracked

During 2015, there was a positive SAM index anomaly, which became increasingly negative toward the end of 2016 (Figure 2a). During the five years considered (2014–2018), maximum monthly EKE values of the STCZ and ABFZ eddy fields were observed during October and November 2015, respectively (Figure 2b). The monthly EKE values at both eddy fields then followed a decreasing trend toward the end of 2016 and 2017, respectively, before gradually increasing (Figure 2b). Compared to the latitudinal position of the SAF during 2014, the front was relatively close to the island throughout the duration of the study (Figure 2c). The PF gradually moved closer to the island over the duration of the study, from an average of 49.8°S in October 2015 to 48.3°S in January 2018, representing approximately 160 km (Figure 2c). Over the three breeding seasons, wind speed within the foraging distribution of the albatrosses was highest in 2015/16 and lowest during 2016/17 (Figure 2d).

### 3.2 Spatial distribution

**FIGURE 3:**
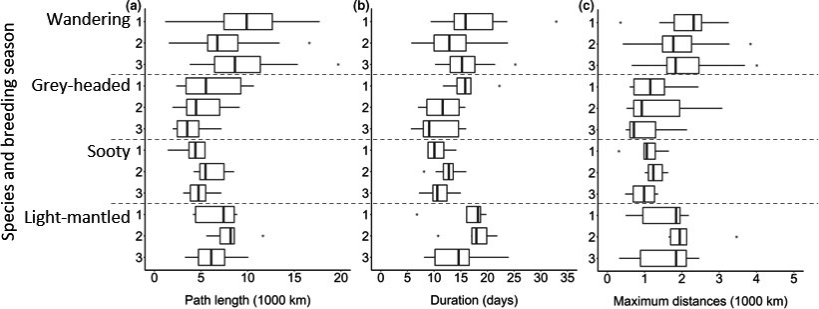
Boxplots of trip parameters: (a) path length, (b) duration, and (c) maximum distance calculated from foraging trips of incubating wandering, grey‐headed, sooty, and light‐mantled albatrosses at Marion Island tracked over three consecutive breeding seasons: (1) 2015/16; (2) 2016/17; and (3) 2017/18. Boxplots show range excluding outliers, upper and lower quartiles, and median with outliers indicated with dots

A significant interaction between year and species was found for all trip parameters (Figure 3; *p* < .01). Post hoc pairwise comparisons revealed that, within species, foraging trips were similar in duration, path length, and maximum distance reached across years for all species (*p* > .05 for all comparisons; Figure 3). However, WA and LMSA foraging trips were significantly longer, and they travelled farther from the island than GHA and SA (Figure 3, Table S1).

**FIGURE 4:**
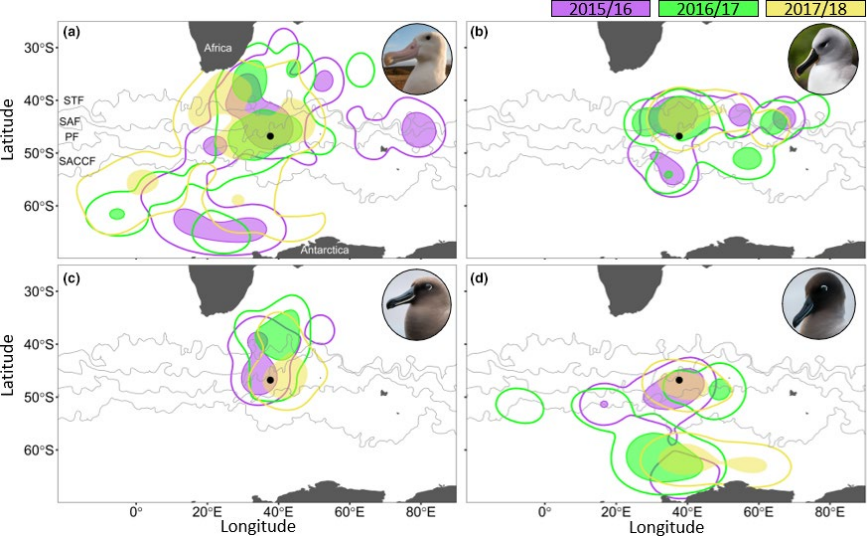
90% (line) and 50% (shaded area) utilization distribution contours of (a) wandering, (b) grey‐headed, (c) sooty, and (d) light‐mantled albatrosses incubating on Marion Island (black dot) over three consecutive breeding seasons (purple: 2015/16, green: 2016/17, and yellow: 2017/18) in relation to the position of subtropical (STF), Sub‐Antarctic (SAF), Antarctic Polar (PF), and Southern Antarctic Circumpolar (SACCF) fronts

Over all three years, the foraging ranges (90% UD) of the study species were generally consistent among years, with >50% overlap for almost all between‐year comparisons within species (Figure 4; Table 1). Overlap of the core foraging areas (50% UD) was more consistent among years for WA (20.8%–28.5%) and GHA (30.5%–35.6%) compared to that of the SA (5.1%–9.1%) and LMSA (0%–25.6%). During 2015/16, compared to other years, WA exhibited an additional core foraging area east of the Kerguelen Plateau (Figure 4a).

**TABLE 1:** Kernel utilization distribution overlap (Bhattacharyya's Affinity) at the 50% and 90% utilization distributions (UD) among three breeding seasons (Y1:2015/16; Y2: 2016/17; and Y3:2017/18) of the four albatross species breeding on Marion Island

Species	Year comparison	At the 50% UD				At the 90% UD			
		Observed overlap	Permuted overlap (mean ± *SD*)	*p*‐Value	*p*‐Value 95% CI	Observed overlap	Permuted overlap (mean ± *SD*)	*p*‐Value	*p*‐Value 95% CI
Wandering	Y1 vs. Y2	25.97	23.12 ± 5.00	.65	0.62–0.68	60.04	54.86 ± 5.48	.83	0.81–0.86
	Y1 vs. Y3	20.77	19.78 ± 5.69	.53	0.50–0.56	53.42	55.53 ± 6.03	.35	0.32–0.38
	Y2 vs. Y3	28.50	28.26 ± 5.14	.50	0.47–0.53	56.37	68.25 ± 4.61	.25	0.22–0.27
Grey‐headed	Y1 vs. Y2	30.35	19.89 ± 8.23	.96	0.94–0.97	66.48	51.46 ± 9.58	.96	0.95–0.97
	Y1 vs. Y3	32.31	28.02 ± 7.71	.66	0.63–0.69	39.49	61.24 ± 8.37	.84	0.82–0.87
	Y2 vs. Y3	35.60	33.41 ± 4.75	.65	0.62–0.68	61.92	63.45 ± 6.36	.38	0.35–0.41
Sooty	**Y1 vs. Y2**	**5.10**	**24.24 ± 8.09**	**.03**	**0.02**–**0.04**	**60.9**	**74.51 ± 5.91**	**.03**	**0.02**–**0.05**
	Y1 vs. Y3	9.05	21.38 ± 8.11	.07	0.06–0.09	52.53	69.57 ± 8.78	.06	0.05–0.08
	**Y2 vs. Y3**	**7.31**	**25.67 ± 8.98**	**.04**	**0.03**–**0.05**	**63.89**	**76.78 ± 5.43**	**.03**	**0.02**–**0.04**
Light‐mantled	Y1 vs. Y2	0.00	13.63 ± 7.41	.08	0.07–0.10	37.19	56.74 ± 10.39	.06	0.04–0.07
	Y1 vs. Y3	25.58	21.67 ± 7.91	.66	0.63–0.69	55.58	62.75 ± 8.35	.20	0.17–0.22
	Y2 vs. Y3	15.74	21.15 ± 7.86	.20	0.18–0.23	50.83	64.84 ± 8.02	.06	0.04–0.07

Note

Significantly different UDs (at *α* = 0.05, in bold) were identified by comparing the real (observed) overlap to a distribution of overlaps from 1,000 permutations of the year labels.

GHA exhibited the highest interannual overlap in their core foraging areas and foraging ranges (Table 1), resulting from consistent foraging along the STCZ (Figures 1b and 4b). However, only during 2015/16 and 2016/17 was foraging evident south‐west of the island within the ABFZ eddy field (Figure 4b). The absence of GHA foraging around the ABFZ eddy field in 2017/18 coincided with the lowest monthly EKE recorded during December when the albatrosses were tracked (Figure 2b; 2015:483.5 cm^−1^ s^−2^; 2016:369.6 cm^−1^ s^−2^; and 2017:329.3 cm^−1^ s^−2^).

Interestingly, even though SA exhibited the most restricted foraging range in terms of longitude (longitudinal range covered: 24.5–53.45°) among all species (WA: −19.5–53.45°; GHA: 22.0–76.2° and LMSA: −10.2–65.0°; Figure 4), they had the lowest degree of overlap between core foraging areas among years (Table 1). In all three years, SA 50% UDs were north of the island; however, during 2015/16 and 2017/18, they extended southward into the Polar Frontal Zone (PFZ), the region between the SAF and PF (Figure 4c).

LMSA consistently foraged south of the island, with 90% UD contours reaching the Antarctic continent in all three years (Figure 4d). Similar to GHA, a core foraging area in 2015/16 was found within the ABFZ eddy field (Figure 4b,d). Like SA, LMSA had a core foraging area within the PFZ in 2017/18 when the PF was farther north than usual (Figures 2c and 4d).

### 3.3 Foraging habitat preference

**FIGURE 5:**
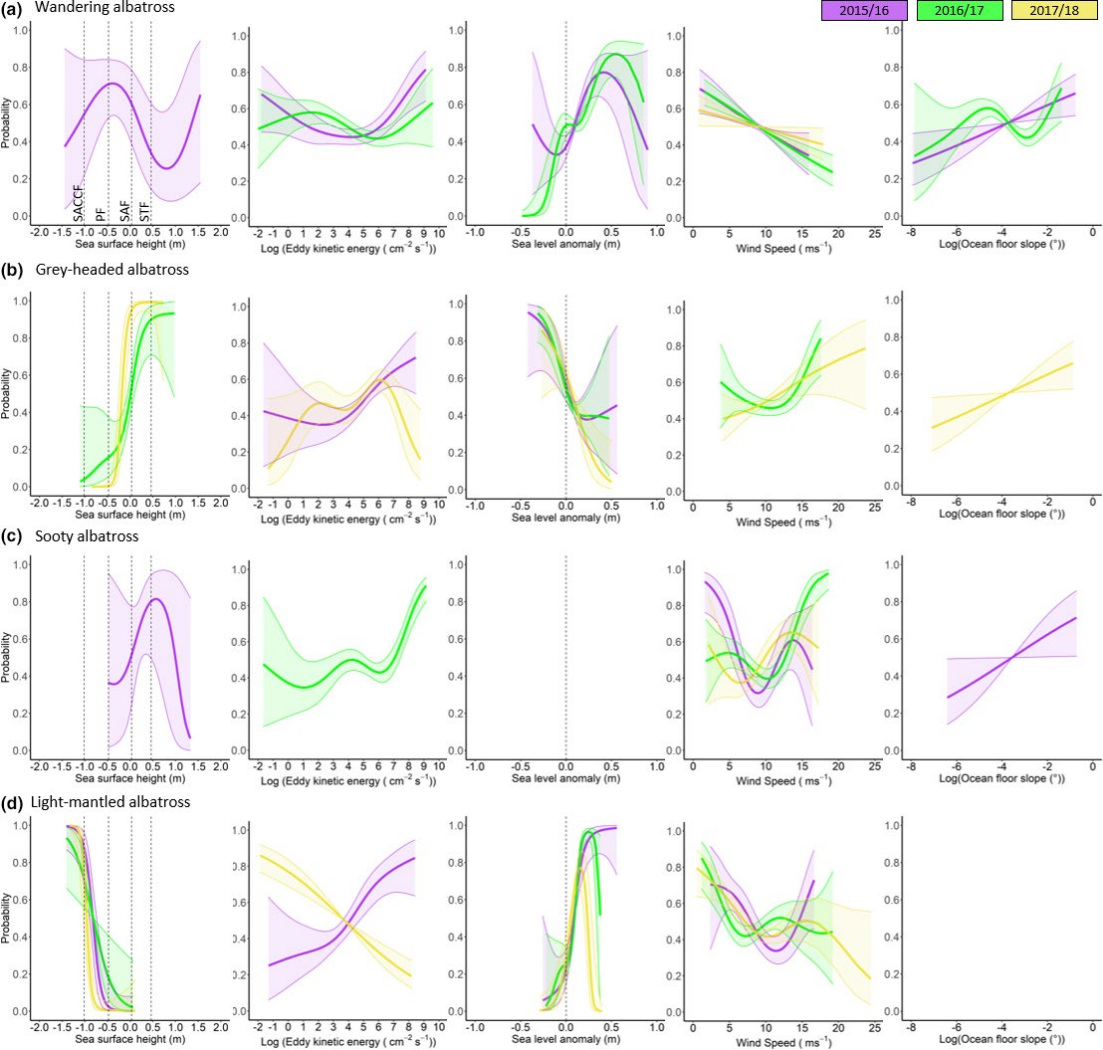
Significant smooths resulting from generalized additive mixed models showing the probability of birds being in foraging (1) and transiting (0) behavioral modes among (a) wandering, (b) grey‐headed, (c) sooty, and (d) light‐mantled albatrosses incubating on Marion Island in relation to, from left to right: sea surface height (SSH) for major fronts in the Southern Ocean indicated by dotted lines (from left to right: South Antarctic Circumpolar (SACCF), Polar (PF), Sub‐Antarctic (SAF) and subtropical (STF) fronts), eddy kinetic energy, sea‐level anomaly, wind speed, and ocean floor slope over three consecutive breeding seasons (purple: 2015/16, green: 2016/17, and yellow: 2017/18)

The probability to switch from transiting to foraging behaviors, in response to a particular suite of environmental variables during a foraging trip, varied across species and years (Figure 5; Table 2). Foraging of WA were significantly correlated to SSH values between the PF and SAF (i.e., PFZ) and above the STF during 2015/16 and below the PF in 2016/17 (Table 2; Figure 5). During both these years, WA were more likely to forage within anticyclonic eddies, evident by foraging behavior being significantly correlated to relatively high EKE and SLA values (Table 2; Figure 5; Figure S4). The first two years (2015/16 and 2016/17) were characterized by a high probability of foraging in areas of steep gradient of the ocean floor slope (Table 2; Figure 5). However, during all three years, foraging of WA was significantly correlated to low wind speeds, whereas transiting was significantly correlated to high wind speeds (Figure 5).

**TABLE 2:** Summary of generalized additive mixed effect model outcomes used to investigate the probability to switch between foraging and transiting behavioral models while at sea in relation to environmental covariates (sea surface height SSH, sea‐level anomaly SLA, eddy kinetic energy EKE, wind speed WIND, and ocean floor slope SLOPE) of wandering, grey‐headed, sooty and light‐mantled albatrosses breeding on Marion Island over three breeding seasons (Year 1:2015/16; Year 2:2016/17; and Year 3:2017/18). Significant relationships are indicated in bold.

Species	Term	edf		Chi. Sq	*p*‐Value	Dev. Expl. (%)
Wandering	**s(SSH): Year 1**	**3.48**	**3.82**	**18.92**	**<.01**	26
	s(SSH): Year 2	2.80	3.3	8.06	.06	
	s(SSH): Year 3	1.00	1.00	1.42	.23	
	**s(EKE): Year 1**	**3.01**	**3.53**	**15.15**	**<.01**	
	**s(EKE): Year 2**	**3.08**	**3.58**	**9.07**	**.03**	
	s(EKE): Year 3	1.15	1.28	0.56	.63	
	**s(SLA): Year 1**	**3.31**	**3.75**	**20.01**	**<.01**	
	**s(SLA): Year 2**	**3.74**	**3.96**	**28.67**	**<.01**	
	s(SLA): Year 3	1.53	1.89	1.74	.32	
	**s(WIND): Year 1**	**1.19**	**1.36**	**10.76**	**<.01**	
	**s(WIND): Year 2**	**1.00**	**1.00**	**23.79**	**<.01**	
	**s(WIND): Year 3**	**1.00**	**1.00**	**4.54**	**.03**	
	**s(SLOPE): Year 1**	**1.00**	**1.00**	**6.99**	**<.01**	
	**s(SLOPE): Year 2**	**3.55**	**3.89**	**19.14**	**<.01**	
	s(SLOPE): Year 3	1.51	1.86	3.58	.22	
Grey‐headed	s(SSH): Year 1	2.51	2.93	4.30	.17	41.3
	**s(SSH): Year 2**	**3.38**	**3.77**	**15.27**	**<.01**	
	**s(SSH): Year 3**	**2.96**	**3.37**	**35.08**	**<.01**	
	**s(EKE): Year 1**	**2.41**	**2.95**	**13.04**	**<.01**	
	s(EKE): Year 2	1.00	1.00	1.26	.26	
	**s(EKE): Year 3**	**3.59**	**3.90**	**13.49**	**<.01**	
	**s(SLA): Year 1**	**2.63**	**3.19**	**9.82**	**.02**	
	**s(SLA): Year 2**	**2.61**	**3.14**	**20.72**	**<.01**	
	**s(SLA): Year 3**	**1.59**	**1.97**	**11.49**	**<.01**	
	s(WIND): Year 1	1.31	1.54	0.85	.41	
	**s(WIND): Year 2**	**2.79**	**3.28**	**11.33**	**.02**	
	**s(WIND): Year 3**	**1.39**	**1.68**	**5.49**	**.03**	
	s(SLOPE): Year 1	1.00	1.00	0.02	.89	
	s(SLOPE): Year 2	2.21	2.76	2.45	.32	
	**s(SLOPE): Year 3**	**1.00**	**1.00**	**5.26**	**.02**	
Sooty	**s(SSH): Year 1**	**2.81**	**3.24**	**9.86**	**.02**	35.9
	s(SSH): Year 2	1.00	1.00	0.30	.58	
	s(SSH): Year 3	2.15	2.60	2.82	.39	
	s(EKE): Year 1	1.00	1.00	0.44	.51	
	**s(EKE): Year 2**	**3.66**	**3.93**	**38.70**	**<.01**	
	s(EKE): Year 3	1.00	1.00	0.86	.35	
	s(SLA): Year 1	1.00	1.00	1.60	.21	
	s(SLA): Year 2	1.96	2.43	2.57	.44	
	s(SLA): Year 3	1.30	1.53	0.80	.44	
	**s(WIND): Year 1**	**3.39**	**3.77**	**22.28**	**<.01**	
	**s(WIND): Year 2**	**3.36**	**3.78**	**30.79**	**<.01**	
	**s(WIND): Year 3**	**2.99**	**3.48**	**14.16**	**<.01**	
	**s(SLOPE): Year 1**	**1.00**	**1.00**	**4.26**	**.04**	
	s(SLOPE): Year 2	1.00	1.00	3.72	.05	
	s(SLOPE): Year 3	1.00	1.00	0.37	.54	
Light‐mantled	**s(SSH): Year 1**	**2.51**	**3.05**	**14.61**	**<.01**	44.5
	**s(SSH): Year 2**	**1.00**	**1.00**	**7.18**	**<.01**	
	**s(SSH): Year 3**	**3.42**	**3.80**	**34.44**	**<.01**	
	**s(EKE): Year 1**	**2.34**	**2.86**	**17.83**	**<.01**	
	s(EKE): Year 2	2.49	3.04	5.08	.17	
	**s(EKE): Year 3**	**1.00**	**1.00**	**34.36**	**<.01**	
	**s(SLA): Year 1**	**3.06**	**3.57**	**23.21**	**<.01**	
	**s(SLA): Year 2**	**3.80**	**3.97**	**78.14**	**<.01**	
	**s(SLA): Year 3**	**3.69**	**3.93**	**44.41**	**<.01**	
	**s(WIND): Year 1**	**2.76**	**3.20**	**17.43**	**<.01**	
	**s(WIND): Year 2**	**3.25**	**3.67**	**13.27**	**<.01**	
	**s(WIND): Year 3**	**3.32**	**3.75**	**27.89**	**<.01**	
	s(SLOPE): Year 1	2.00	2.50	2.61	.46	
	s(SLOPE): Year 2	1.00	1.00	0.002	.96	
	s(SLOPE): Year 3	2.19	2.63	1.75	.42	

The significant correlations between GHA foraging locations and high SLA and EKE values suggest that this species foraged within cyclonic eddies in all three years (Table 2; Figure 5). However, during 2016/17, although the GHA foraging behavior was significantly explained by SLA, it was not by EKE (Table 2). In addition, during the last two years of the study, foraging behavior of GHA was significantly correlated to SSH values characteristic of areas to the north of the PF and SAF, respectively, as well as high wind speeds (Table 2; Figure 5).

Surprisingly, the foraging areas of SA were poorly correlated with any of the environmental variables (Table 2; Figure 5). In 2015/16, foraging was only significantly correlated to SSH values along the SAF and STF, whereas in 2016/17, foraging locations were correlated with high EKE values (Table 2). During 2017/18, foraging was only correlated with steep gradients of the ocean floor (>4,000 m; Table 2; Figure 5). However, foraging of SA was significantly correlated to low wind speeds in 2015/16 and high wind speeds in 2016/17 and 2017/18 (Figure 5).

LMSA foraging locations were significantly correlated to low SSH values and positive SLA values over all three years (Table 2; Figure 5). In addition, during 2015/16, LMSA were significantly more likely to forage in areas with high EKE values whereas during 2017/18 they were significantly more likely to forage in areas with low EKE values (Table 2). Over all three years, the LMSA were significantly more likely to forage in low wind speeds but also within high wind speeds during 2015/16 (Table 2; Figure 5).

### 3.4 Variability in foraging habitats

**FIGURE 6:**
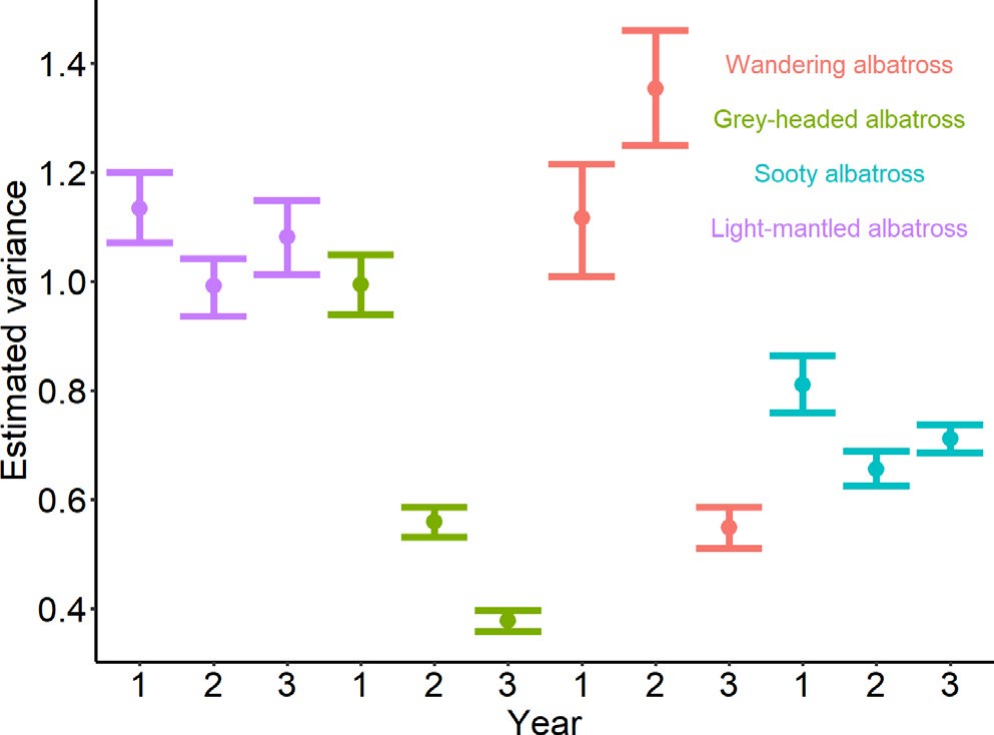
FI Interindividual estimated variance components for scaled environmental variables (sea surface height, eddy kinetic energy, sea‐level anomaly, and ocean floor slope) characterizing the foraging habitat for the four albatross species incubating at Marion Island between three breeding seasons (1:2015/16, 2:2016/17, and 3:2017/18). Filled circles show the estimated variance, and the bars show the credible intervals around these estimates

The multivariate Bayesian mixed model revealed that the variance in environmental variable values (i.e., SSH, EKE, SLA, wind speed, and ocean floor slope) experienced by birds when foraging differed between years and species (Figure 6). Unexpectedly, during the first two years, SA exhibited a much greater variance in foraging habitat used compared to all other species. Foraging of individual SA occurred at a large range of environmental variable values, especially compared to GHA and LMSA (Figure S6). Similarly, the consistently high variance in habitat use exhibited by WA could be attributed to individuals foraging across a large range of environmental variable values (Figures S6and S7). Both GHA and LMSA showed decreasing variance in environmental variable values at foraging locations over the three years (Figure 6). This corresponded to both species having a higher density of foraging locations at specific values of SSH and SLA during 2017/18 compared to other years (Figure S7) as well as individuals foraging over relative narrow ranges of environmental variable values (Figure S6).

## 4 DISCUSSION

We have investigated the response in foraging behavior of breeding sympatric albatross species to environmental variability using concurrently collected tracking data over three consecutive years. Results from WA provided strong support for our “nomad hypothesis” as individuals exploited an extensive area and demonstrated large variance in interindividual foraging habitat use. By comparison, the smaller albatrosses provided partial evidence toward two of our hypotheses. All three smaller albatrosses showed consistency in spatial distribution, supporting the “faithful hypothesis”. However, the interannual differential use of foraging habitat by GHA and LMSA also provided partial support for our “flexible hypothesis”. Furthermore, the high variance in habitat use by SA indicated that this species was not faithful to a particular foraging habitat thus providing partial evidence toward our “nomad hypothesis”. Overall, our results provide clear evidence of flexibility in albatross foraging behavior in response to environmental variability.

### 4.1 The wandering albatross: a true nomad?

Throughout the three years, WA were consistently distributed across an extensive area and their foraging behavior was associated with low wind speeds whereas they were more likely to transit in high wind speeds. There was evidence of WA foraging at several oceanographic features known to have elevated productivity, including, but not restricted to, the edge of the Agulhas Bank and Agulhas retroflection, eddies in the proximity of the STCZ and ABFZ and within major fronts (Figure 1, Figure S5). This wide‐ranging spatial distribution and foraging habitat use has previously been described as typical for WA breeding at Marion Island (Nel et al., 2002) and elsewhere (Cleeland et al., 2019; Phillips et al., 2008; Weimerskirch, 2007). These birds cover large areas by employing a looping foraging strategy (Weimerskirch, 2007), frequently transiting between bouts of foraging. This differs from a commuting foraging strategy, where birds move directly toward a foraging area then return directly to their colonies (Weimerskirch, 2007).

Previously, WA have been observed to segregate foraging ranges among sexes and age classes (de Grissac, Bartumeus, Cox, & Weimerskirch, 2017; Lecomte et al., 2010; Nel et al., 2002; Weimerskirch, Gault, & Cherel, 2005; Weimerskirch et al., 2012). Incubating female WA typically forage in warmer, more northern waters than males (Nel et al., 2002; Weimerskirch et al., 2012). Furthermore, younger incubating males usually forage north of the PF, farther north than older incubating males, which regularly forage south of 60°S (Lecomte et al., 2010). This suggests that WA are not true nomads but that their foraging behavior is structured to limit intraspecific competition for prey. However, the consistent use of multiple habitats across individuals can be considered nomadic feeding behavior, especially compared to those species that commute to favored foraging areas (Weimerskirch, 2007).

Weimerskirch et al. (2012) showed that as a response to an increasingly more positive SAM index and associated increases in wind speeds and southward migration of the Southern Ocean's westerly wind belt, the foraging ranges of both male and female WA breeding on the Crozet Archipelago are shifting southward. This study presents the first record of WA incubating on Marion Island traveling south of 60°S, with ~25% of tracked individuals (*n* = 11) traveling south of 60°S and 34% south of 55°S. Nel et al. (2002) found that only one of 11 incubating WA on Marion Island tracked during the late 1990s briefly ventured below 55°S. However, Weimerskirch et al. (1993) observed incubating WA from Possession Island (Crozet Archipelago) foraging south of 60°S, so it is hard to know whether the Marion Island population has shifted their foraging range farther south since 1990s, or whether this result is simply an artifact of the greater number of individuals tracked compared to Nel et al. (2002).

### 4.2 Grey‐headed and light‐mantled albatrosses: flexible faithfulness

Both GHA and LMSA exploited two different foraging habitats, with their use differing among years. Over the three years, GHA consistently foraged in and around eddies in the proximity of the STCZ; however, only during 2015/16 and 2016/17 was there evidence of GHA foraging south‐west of the island, within the ABFZ eddy field. The complete absence of the GHA in this area during 2017/18 corresponded to the lowest monthly EKE recorded for the ABFZ during December, when all small albatrosses were tracked (Figure 2). Previously, GHA incubating on Marion Island have been inferred to forage within both the STCZ and the ABFZ eddy fields (Nel et al., 2001). The monthly EKE for the ABFZ eddy field was 444.9 cm^−1^ s^2^ during December 1997, when GHA were tracked by Nel et al. (2001), which is similar to the monthly EKE during years when we found GHA foraging with the ABFZ eddy field. Together with the findings of Nel et al. (2001), we show that incubating GHA only forage within the ABFZ eddy field when there is high EKE, indicative of eddy presence.

Major frontal systems are known to be important foraging habitats of breeding GHA from other colonies (Cleeland et al., 2019; Scales et al., 2016; Xavier, Croxall, Trathan, & Wood, 2003). As at Marion Island, differential use of two habitats in response to environmental variability has been found for GHA rearing chicks on Bird Island, South Georgia (Xavier, Croxall, Trathan, et al., 2003). In years with “good” environmental conditions, Bird Island GHA were more likely to forage at the PF and feed on cephalopods, resulting in higher breeding success (Xavier, Croxall, Reid, et al., 2003; Xavier, Croxall, Trathan, et al., 2003; Xavier et al., 2013). By comparison, in years when GHA mainly foraged on krill in Antarctic waters, they experienced poorer breeding success. However, although the proportion of cephalopod, fish, and krill within the diet of the GHA rearing chicks at Marion Island remains relatively consistent between years, there is substantial interannual variance in the species composition of these taxa (Connan, McQuaid, Bonnevie, Smale, & Cherel, 2014; Hunter & Klages, 1989; Nel et al., 2001; Richoux, Jaquemet, Bonnevie, Cherel, & McQuaid, 2010). The findings of these studies highlight the flexibility in GHA foraging behavior across its range. However, contrasting population trends at Marion (increasing; BirdLife International, 2020) and Bird (steadily decreasing; Pardo et al., 2017; Poncet et al., 2017) islands suggest that resilience of this species to climate change will be population specific, despite their foraging flexibility.

Most LMSA from Marion Island foraged in two areas: within the PFZ or well south of the Southern Antarctic Circumpolar (SACCF), close to the Antarctic continent. However, two of 22 tracked birds travelled west, and five remained relatively close to the island north of 55°S. Interestingly, LMSA only foraged within the ABFZ eddy field in 2015/16, when monthly EKE for this area was at its highest (Figure 2; Figure S4). Unlike GHA, individual LMSA that travelled south often foraged within both key foraging habitats during a single trip. The use of a habitat close to their colonies as well as far south, along the coast of Antarctica, seems to be typical behavior of LMSA across their distribution (Cleeland et al., 2019; Delord et al., 2013; Phillips et al., 2005). However, LMSA breeding on the Crozet and Kerguelen archipelagos increase their use of their distant Antarctic foraging grounds during the more energy demanding chick‐rearing stage, relative to during incubation (Delord et al., 2013). In addition, even though Phillips et al. (2005) found evidence of LMSA foraging in two different habitats, they inferred that LMSA take little or no prey in the PFZ due to the low incidence of sub‐Antarctic cephalopod species in their diet. This does not seem to be the case for LMSA breeding on Marion Island. Previous investigations into the diet composition of LMSA breeding at Marion Island found similar proportions of sub‐Antarctic and Antarctic cephalopod species (Connan et al., 2014; Cooper & Klages, 1995). This suggests that both of these habitats are important foraging grounds for this population of LMSA.

### 4.3 Sooty albatross: faithful nomads

The interannual variability in foraging behavior of SA was perhaps the most unexpected. Most individuals transited between foraging bouts all along their tracks, without evidence of direct movement toward a terminal point. This was evident in the very high variance in the values of environmental variables used while foraging (Figure 6) and large spread of values experienced by individuals, although only during the initial two years of the study (Figure S5). The low variance during the last year corresponded to a greater number of individuals remaining within the PFZ and SAF and not traveling north of the STF. These results suggest that SA are foraging habitat generalists that are particularly flexible in their foraging behavior. Across populations of SA, whether colonies are north (Tristan and Amsterdam islands) or south of the STF (Gough, Crozet, and Marion islands), core foraging areas of SA are situated just north of the STF (this study; Delord et al., 2013; Pinaud & Weimerskirch, 2007; Schoombie, Dilley, Davies, Glass, & Ryan, 2017). Whether SA breeding elsewhere are “faithful nomads” is difficult to say, as previous studies involving this species have not focussed on consistency in habitat use (Delord et al., 2013; Pinaud & Weimerskirch, 2007; Schoombie et al., 2017).

### 4.4 THE IMPORTANCE OF THE SUBTROPICAL CONVERGENCE TO INCUBATING ALBATROSSES ON MARION ISLAND

Over all three years of the study, three albatross species (WA, GHA, and SA) foraged within and around eddies originating at the STCZ (Figure S4). Although this oceanographic feature does exhibit some inter and intra‐annual variability, it consistently represents the area of highest EKE within the Southern Ocean (Meredith & Hogg, 2006). Thus, the STCZ appears to represent a predictable foraging area for predators breeding at Marion Island. In agreement, GHA (Nel et al., 2001) and SA (Schoombie et al., 2017) breeding at Marion Island and other seabirds breeding on the neighboring Crozet Archipelago (Pinaud & Weimerskirch, 2007; Weimerskirch et al., 2005) have been found to forage within and around eddies produced at the STCZ. Interestingly, the only species not utilizing this feature or not foraging north of the island (LMSA) is the only albatross species that has been declining at both Marion Island (Ryan, Jones, Dyer, Upfold, & Crawford, 2009; Schoombie et al., 2017) and the Crozet Archipelago over the past decade (Weimerskirch, 2018; Weimerskirch et al., 2018). Inchausti et al. (2003) found that contrasting trends in breeding success of seabirds breeding on Crozet and Kerguelen Archipelagos correlated to the different species’ foraging distribution. Seabird species which foraged south of the PF experienced lower breeding success during climatically anomalous years, whereas the breeding success of seabirds which foraged in subtropical water was higher.

### 4.5 Resilience to environmental change

Behavioral plasticity plays a key role in the ability of organisms to adjust to environmental variability and change (Beever et al., 2017). Here, we showed that incubating WA, GHA, SA and LMSA are flexible in their spatial distribution and foraging habitat use either by exhibiting behaviors which are typical of foraging habitat generalists or the ability to switch between habitats or a combination of the two. Although it is difficult to quantify behavioral plasticity directly, this flexibility in foraging habitat use and distribution indicates that species in this study exhibited a degree of foraging behavioral plasticity even though there was a low per year sample size. WA are likely to be at least partially buffered against changing environmental conditions due to their broad use of habitats, especially since WA breeding success is either positively correlated to environmental variability or change (Inchausti et al., 2003; Weimerskirch et al., 2012) or is not correlated at all (Lewis, Nussey, Wood, Croxall, & Phillips, 2012; Rolland, Weimerskirch, & Barbraud, 2010). These are encouraging findings, as it potentially indicates some resilience of albatrosses to environmental variability and change (Beever et al., 2017; Kappes et al., 2010).

In many instances, fluctuations in albatross breeding success have been related to environmental conditions, with years of anomalous climatic conditions often resulting in decreased breeding success (Croxall et al., 1999; Inchausti et al., 2003; Kappes et al., 2010; Nevoux, Forcada, Barbraud, Croxall, & Weimerskirch, 2010; Pinaud, Cherel, & Weimerskirch, 2005; Thorne et al., 2015; Veit, Silverman, & Everson, 1993; Weimerskirch et al., 2012). In all instances where breeding success was negatively correlated to environmental variability, albatrosses had to increase their energy expenditure to either track preferred habitats (Kappes et al., 2010; Thorne et al., 2015) or switch to exploit other habitats (Pinaud et al., 2005). Similarly, an increase in breeding success of the WA breeding at the Crozet Archipelago was correlated with a decrease in energy expenditure linked to increased wind speeds that resulted in faster flight speeds and shorter foraging trips (Weimerskirch et al., 2012). The lack of a difference in trip parameters between years for all species in this study suggests that interannual changes in foraging habitat and distribution did not require increased foraging effort or energy. However, Thorne et al. (2015) found that during a strong La Nina event, foraging trip parameters of incubating Laysan albatrosses *Phoebastria immutabilis* did not change compared to pre‐La Nina conditions. Nonetheless, once chicks hatched parents travelled further than previously recorded during the brooding stage, resulting in lowered breeding success during the La Nina event.

It is therefore important to note that this study only tracked incubating individuals. During this breeding stage, foraging trip duration and foraging range is limited by the fasting capabilities of their partners, which is much greater than that of a newly hatched and growing chick. Nel et al. (2001) showed that once a GHA egg hatches, adult GHA breeding at Marion Island no longer travel to the STCZ and all foraging activity is restricted around prey aggregating mesoscale eddies in the lee of the ABFZ. As a result of climate change, southward shifts of the major frontal zones within the Southern Ocean has been predicted (Kim & Orsi, 2014; Wilson et al., 2016 but see Chapman et al., 2020; Meijers et al., 2012). Asdar (2018) recently showed that if there is a 1° latitudinal shift, either north or south, in the position of the PF, the PF will no longer interact with the ABFZ, producing fewer eddies. This could result in the loss of an important and predictable foraging habitat for GHA brooding chicks at Marion Island. Interannual variability in foraging habitat during the brooding stage has not been studied for any of the other albatrosses on Marion Island. It is thus important to expand on the findings of this study and investigate the foraging behavior of albatrosses during brooding, especially how they respond to environmental variability during this time.

Future climate scenarios suggest an increase in SAM index values over the next century in response to climate change (Gillett & Fyfe, 2013). As this is associated with an increase in wind speed in the Southern Ocean, it is likely that this will have a positive influence on the number of prey aggregating mesoscale eddies (Meredith & Hogg, 2006; Morrow et al., 2010). However, further investigations are needed to understand the regional effects of increasingly positive SAM indices on existing eddy fields which act as important foraging habitat to the seabirds and seals breeding on Marion Island, such as the STCZ and ABFZ eddy fields. Furthermore, as shown by Weimerskirch et al. (2012), increased wind speeds could increase the foraging efficiency of albatrosses by increasing their flying speeds while transiting and thus decreasing their overall trip durations. However, due to highly positive SAM indices predicted by 2080, the wind speed south of Marion Island is expected to increase, whereas wind speed to the north of the island is expected to decrease (Weimerskirch et al., 2012). This may have a negative impact on those flying seabird species which forage to the north of Marion Island.

### 4.6 Study limitations and future priorities

A limitation to our study is that variation in habitat use between sexes was not taken into consideration. Previously, segregation of foraging areas between the sexes of WA incubating on Possession Island (Lecomte et al., 2010; Weimerskirch et al., 2012) and GHA incubating on Bird Island (Phillips et al., 2004) has been shown. Due to our relatively small sample size of birds sampled each year (Table S1), it is possible that yearly differences we have observed were influenced by an unbalanced number of males and females sampled each year. Future research on these study species at Marion Island would benefit from considering intrinsic (e.g., sex and age) and extrinsic (e.g., con‐ and interspecific interactions) influences on foraging habitat preference and spatial distribution.

### 4.7 Conclusions

Our findings highlight foraging plasticity in four albatross species in response to environmental variability. Future investigations should include other breeding stages during the annual cycles of the albatrosses, coupled with demographic studies to further our understanding of behavioral plasticity and population responses. Furthermore, as this study has investigated the response in foraging behavior of albatrosses at the population level, a future logical progression would be to investigate individual variability in foraging behavior as well as a comparison between populations with different external stressors (e.g., fisheries, Barbraud et al., 2012; Grémillet et al., 2018). This study, however, demonstrates the importance of elucidating links between oceanography and behavior to understand the response of seabirds to climate variability and change.

## CONFLICT OF INTERESTS

Authors have no conflicts of interest to declare.

## AUTHOR CONTRIBUTION

**Tegan Carpenter‐Kling:** Data curation (lead); Formal analysis (lead); Methodology (lead); Visualization (lead); Writing‐original draft (lead). **Ryan R. Reisinger:** Formal analysis (supporting); Methodology (supporting); Supervision (supporting); Writing‐review & editing (equal). **Florian Orgeret:** Formal analysis (supporting); Methodology (supporting); Supervision (supporting); Writing‐review & editing (equal). **Maëlle Connan:** Conceptualization (supporting); Supervision (supporting); Writing‐review & editing (equal). **Peter G. Ryan:** Conceptualization (supporting); Funding acquisition (supporting); Writing‐review & editing (equal). **Azwianewi Makhado:** Conceptualization (supporting); Funding acquisition (supporting); Writing‐review & editing (equal). **Kim L. Stevens:** Data curation (supporting); Writing‐review & editing (equal). **Pierre A. Pistorius:** Conceptualization (lead); Funding acquisition (lead); Supervision (lead); Writing‐review & editing (equal).

## ETHICAL APPROVAL

The project received ethics clearance from the Nelson Mandela University Animal Ethics Committee (A14‐SCI‐ZOO‐012).

## Supporting information

Supplementary Material

## Data Availability

Data used in this research are publicly available at https://doi.org/10.5061/dryad.q2bvq83h0.
