# Supplementary material for: Foraging in a dynamic environment: Response of four sympatric sub‐Antarctic albatross species to interannual environmental variability
Source: Ecol Evol. 2020 Sep 21;10(20):11277–95. doi: 10.1002/ece3.6766 (PMC7593157; doi:10.1002/ece3.6766)
Supplement: Supplementary file 1 — Supplementary Material [file ECE3-10-11277-s001.docx]

**Supplementary Material**

**Response of four sympatric sub-Antarctic albatross species to environmental variability**

Tegan Carpenter-Kling, Ryan R. Reisinger, Florian Orgeret, Maëlle Connan, Kim L. Stevens, Peter G. Ryan, Azwianewi Makhado and Pierre A. Pistorius

| Common name | Year | Tracks (n) | Locations (n) | Start date | End date | Path length (km) | Duration (day) | Maximum distance (km) |
| --- | --- | --- | --- | --- | --- | --- | --- | --- |
| Wandering albatross | 2015/16 | 9 | 2943 | 2016/01/22 | 2016/03/02 | 9619.7 ± 4821.4 | 18.4 ± 7.0 | 2220.7 ± 915.7 |
|  | 2016/17 | 16 | 2916 | 2017/02/21 | 2017/04/01 | 7529.6 ± 3935.2 | 13.3 ± 5.2 | 1854.5 ± 895.8 |
|  | 2017/18 | 20 | 7453 | 2018/02/02 | 2018/03/12 | 9238.2 ± 3877.8 | 15.7 ± 4.0 | 2055.8 ± 919.9 |
| Grey-headed albatross | 2015/16 | 5 | 1832 | 2015/11/29 | 2015/12/27 | 4084.4 ± 1659.1 | 16.2 ± 3.9 | 1288.5 ± 743.4 |
|  | 2016/17 | 11 | 3038 | 2016/11/27 | 2016/12/24 | 6061.0 ± 1592.6 | 11.7 ± 3.3 | 1356.4 ± 901.8 |
|  | 2017/18 | 10 | 2533 | 2017/11/25 | 2017/12/14 | 4788.7 ± 1328.5 | 10.6 ± 4.0 | 970.9 ± 524.9 |
| Sooty albatross | 2015/16 | 5 | 1022 | 2015/11/26 | 2015/12/16 | 2741.5 ± 1274.6 | 10.7 ± 2.3 | 1057.4 ± 490.6 |
|  | 2016/17 | 10 | 2950 | 2016/11/23 | 2016/12/18 | 1994.3 ± 480.6 | 12.4 ± 2.1 | 1278.7 ± 236.7 |
|  | 2017/18 | 8 | 2085 | 2017/11/22 | 2017/12/13 | 2179.0 ± 800.1 | 11.0 ± 2.5 | 954.7 ± 349.6 |
| Light-mantled albatross | 2015/16 | 5 | 1250 | 2015/11/28 | 2016/01/02 | 6675.1 ± 2269.4 | 15.9 ± 5.3 | 1484.3 ± 724.9 |
|  | 2016/17 | 6 | 1517 | 2016/11/26 | 2016/12/29 | 8146.7 ± 2056.4 | 17.6 ± 3.8 | 2132.1 ± 687.2 |
|  | 2017/18 | 11 | 3646 | 2017/11/24 | 2018/01/05 | 6304.2 ± 2160.3 | 14.1 ± 4.7 | 1546.3 ± 762.1 |

**Table S1.** Summary of tracks recorded via GPS data loggers deployed on incubating wandering, grey-headed, sooty and light-mantled albatrosses on Marion Island over three breeding seasons (2015/16-2017/18) and corresponding trip parameters. Values reported are mean ± SD.

**Table S2.** Environmental variables: spatial and temporal resolutions and source

| Variable | Abbreviation | Spatial resolution | Temporal resolution | Source |
| --- | --- | --- | --- | --- |
| Sea surface height (m) | SSH | 0.25° | Daily | CMEMS^a^ |
| Sea level anomaly (m) | SLA | 0.25° | Daily | CMEMS^a^ |
| Eddy kinetic energy | EKE | 0.25° | Daily | CMEMS ^a, b^ |
| Wind speed |  | 0.25° | Daily | CERSAT ^c^ |
| Bathymetry (m) |  | 0.02° | - | GEBCO ^d^ |
| ^a^ Ssalto/Duacs produced and distributed by the Copernicus Marine Environment Monitoring Service (CMEMS, http://marine.copernicus.eu) | | | | |
| ^b^ Downloaded as geostrophic velocities (U and V components) and computed as EKE = 0.5 x (U^2^ + V^2^) | | | | |
| ^c^ Downloaded from CERSAT web portal (http://www.ifremer.fr/cersat) | | | | |
| ^d^ General Bathymetric Chart of the Oceans hosted by the British Oceanographic Data Centre (http://www.gebco.net/data_and_products/gridded_bathymetry_data/) | | | | |

**Table S3.** Sea surface heights (SSH) used to identify major fronts within the Southern Ocean, following the methods of Rintoul (2009a, b) and Swart et al. (2010)

| Front | Sea surface height (m) | | |
| --- | --- | --- | --- |
|  | Northern boundary | Middle | Southern boundary |
| sub-Tropical | 0.5700 | 0.4600 | 0.3500 |
| sub-Antarctic | 0.2400 | 0.0300 | -0.1700 |
| Antarctic Polar | -0.3000 | -0.4800 | -0.6300 |
| South Antarctic Circumpolar | -0.9430 | -1.0157 | -1.0884 |

**Figure S4.** a) Bounding boxes of areas used to investigate monthly variability in eddy kinetic energy of two major eddy fields within the Southern Ocean: sub-Tropical Convergence Zone (STCZ) and the Andrew Bain Fracture Zone (ABFZ) and b) graphical representation of bounding boxes overlaying a map of eddy kinetic energy, black dot represents the Prince Edward Archipelago.


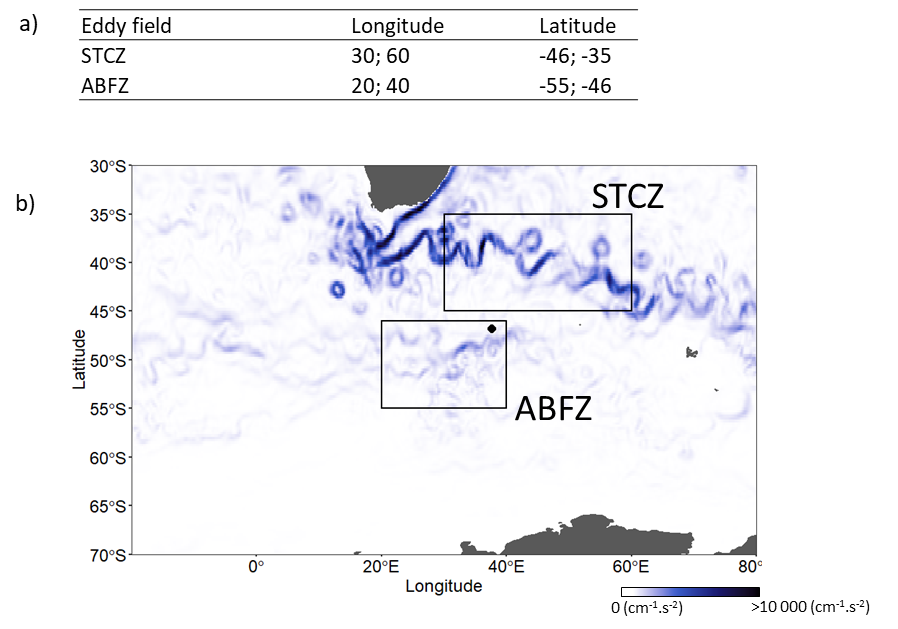


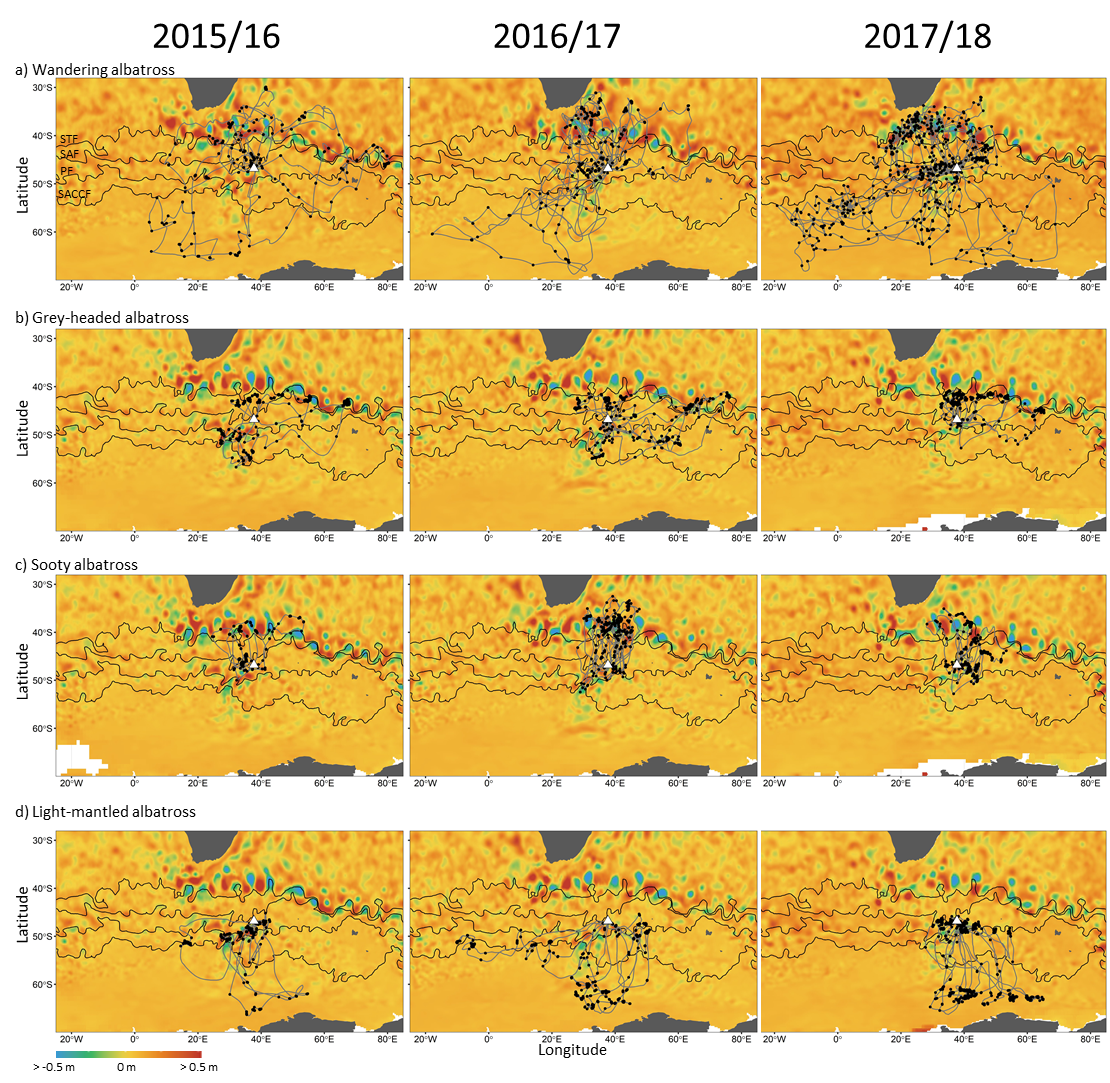
**Figure S5.** GPS tracks and locations identified as foraging by a Bayesian space state model of a) wandering, b) grey-headed, c) sooty and d) light-mantled albatrosses incubating on Marion Island over three breeding seasons (left to right: 2015/16; 2016/17 and 2017/18) overlain on maps of sea level anomaly which was averaged over the time period of the tracks shown on the respective map. Breeding season specific December positions of sub-Tropical (STF), sub-Antarctic (SAF), Antarctic Polar (PF) and Southern Antarctic Circumpolar (SACCF) fronts are shown as black lines and Marion Island (white triangle).

**
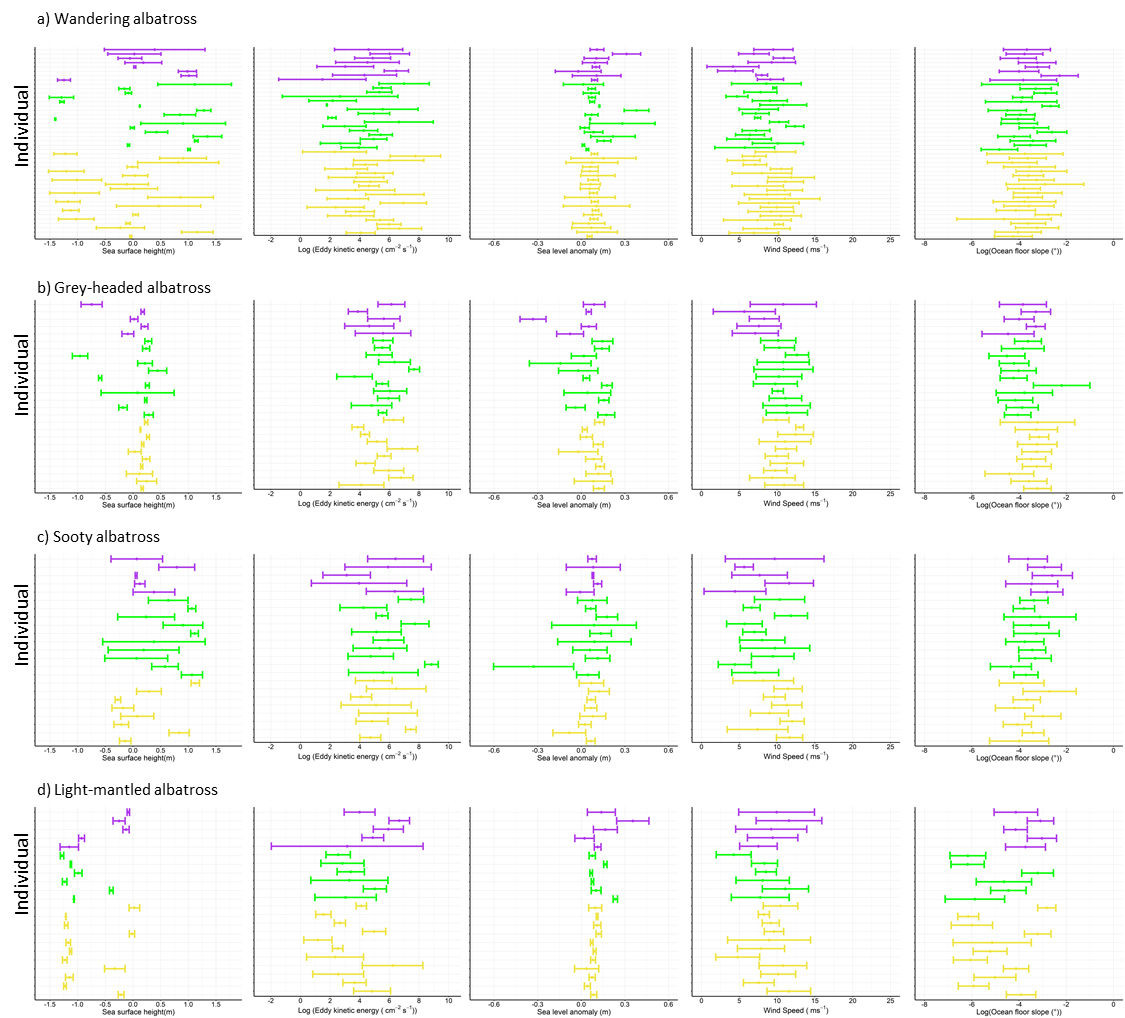
Figure S6**. Median and median absolute deviance of environmental variables under GPS locations along the trajectories of incubating a) wandering, b) grey-headed, c) sooty and d) light-mantled albatross which were identified as the birds being in a foraging behavioural mode. Data are shown for breeding seasons: 2015/16 (purple), 2016/17 (green) and 2017/18 (yellow).


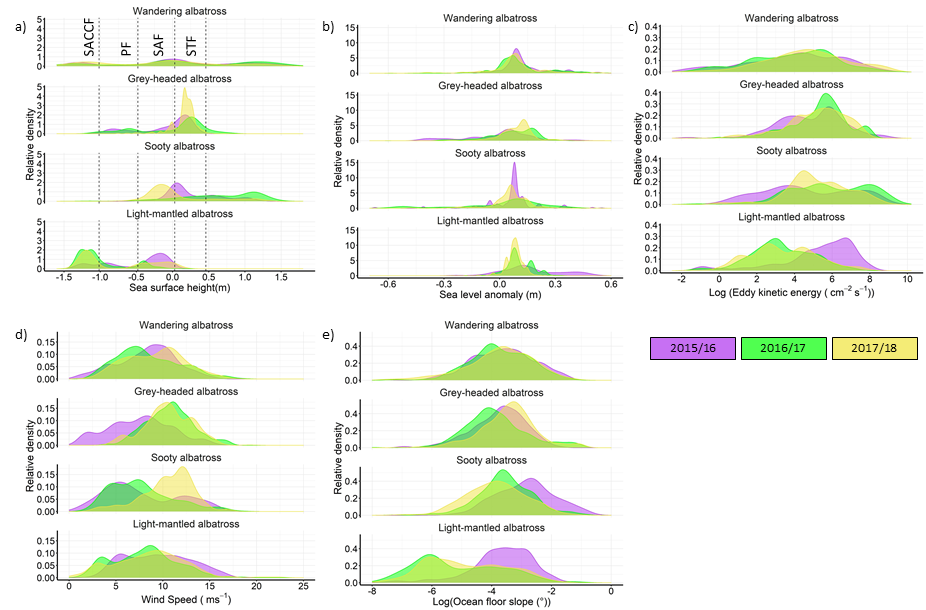


Figure S7. Density plots of a) sea surface height, b) sea level anomaly, c) eddy kinetic energy, d) wind speed and e) ocean floor slope at GPS locations along the trajectories of albatrosses breeding at Marion Island which were identified as the bird most likely being in a foraging behavioural mode. Densities are shown separately for each year: 2015/16 (purple), 2016/17 (green) and 2017/18 (yellow).
